# An Overview of Buckwheat—A Superfood with Applicability in Human Health and Food Packaging

**DOI:** 10.3390/plants14142200

**Published:** 2025-07-16

**Authors:** Alexandra Andreea Lițoiu, Adriana Păucean, Claudiu Lung, Alexandru Zmuncilă, Maria Simona Chiș

**Affiliations:** 1 Department of Food Engineering, Faculty of Food Science and Technology, University of Agricultural Sciences and Veterinary Medicine Cluj-Napoca, 3–5 Manăștur Street, 400372 Cluj-Napoca, Romania; alexandra-andreea.litoiu@student.usamvcluj.ro (A.A.L.); adriana.paucean@usamvcluj.ro (A.P.); alexandru.zmuncila@student.usamvcluj.ro (A.Z.); 2 Department of Condensed State Physics and Advanced Technologies, Faculty of Physics, Babes-Bolyai University, Mihail Kogalniceanu Street, 400372 Cluj-Napoca, Romania; claudiu.lung@ubbcluj.ro

**Keywords:** buckwheat, bioactive compounds, *Fagopyrum tataricum* L., *Fagopyrum esculetum* Moench health benefits, sustainability

## Abstract

Buckwheat, a dicotyledonous pseudocereal from the Polygonaceae family, has emerged as a crop of scientific and industrial interest due to its exceptional phytochemical profile, adaptability to different environments, and minimal agronomic input requirements. This paper aims to highlight the proximate composition (carbohydrates, protein, dietary fiber, lipids, starch, vitamins, and minerals) of the buckwheat principal species, *Fagopyrum esculentum* Moench (common buckwheat) and *Fagopyrum tataricum* (L.) Gaertn (Tartary buckwheat). Other bioactive compounds, including flavonoids (e.g., rutin, quercetin), phenolic acids, and anthocyanins, were emphasized, together with their influence on human health. These constituents confer a broad range of biological activities such as anti-inflammatory, antimicrobial, antidiabetic, antihypertensive, and hypoglycemic effects. Moreover, buckwheat is inherently gluten-free, making it a valuable alternative in formulations targeting gluten-sensitive populations. Finally, the review addresses the possibility of using starch buckwheat as a raw material in starch-based films. Further research is needed to elucidate the potential of buckwheat starch as a viable material for the development of biodegradable food packaging films.

## 1. Introduction

Buckwheat is a pseudocereal that belongs to the Polygonaceae family and *Fagopyrum* genus. The most extensively cultivated species within the *Fagopyrum* genus are *Fagopyrum esculentum* Moench (common buckwheat) and *Fagopyrum tataricum* (L.) Gaertn (Tartary buckwheat), owing to their broad agroecological adaptability and widespread global distribution [1]. Buckwheat (*Fagopyrum* spp.) is predominantly utilized as a pseudocereal, with its seeds consumed in whole or milled to produce flour, which is further used in various food applications. Additionally, its inflorescences are highly valued in apiculture and provide an excellent source of honey production [2].

Pseudocereals are rich in bioactive compounds with balanced nutritive properties [3,4]. Moreover, pseudocereals are used in human nutrition, but buckwheat (*F. esculentum* Moench) and Tartary buckwheat (*F. tataricum* (L.) Gaertn) are the most common species used in the food industry [3]. Crops’ nutritional and therapeutic value should be included in food resources in order to combat hidden hunger with contemporary methods [5]. Moreover, pseudocereals contribute to enhanced agricultural productivity, thereby increasing the availability and diversifying the range of raw materials utilized in food production systems [6]. Additionally, the formulation of value-added food products with functional attributes that meet the nutritional needs of the world should be facilitated in order to advance the UN Agenda 2030’s main objectives of eradicating hunger, guaranteeing food security, enhancing nutrition, and advancing sustainable agriculture [7]. Quinoa (*Chenopodium quinoa* Willd.), amaranth (*Amaranthus caudatus* L.; *A. cruentus*; A. *hypochondriacus*), and buckwheat (*Fagopyrum esculentum* Moench.) are the most widely recognized pseudocereals [5].

On the other hand, pseudocereals are considered to be underexploited, which is defined as food sources that historically constituted integral components of traditional diets across various populations but were progressively displaced during the early 20th century due to the global homogenization of food systems and the dominance of major staple crops [8].

The consumption of buckwheat has been associated with the prevention and mitigation of various chronic non-communicable diseases, including obesity, hypertension, and cardiovascular disorders, due to its favorable nutritional profile and bioactive compound content [9]. Current research on buckwheat, both in vivo and in vitro, confirms its potential health benefits, including antitumor, anti-inflammatory, antioxidant, and hepatoprotective effects [3,10]. Buckwheat’s bioactive components may help prevent several illnesses, including diabetes, high blood pressure, cancer, glycemic disorders, and more [3]. Transitionally, buckwheat has been used in the treatment of diabetes and cancer. It is also known for its various biological properties, including antibacterial, antioxidant, and anti-aging effects. It is a crop recognized for its exceptional nutritional profile and high concentration of bioactive compounds, which contribute to its functional and health-promoting properties [9].

The food industry is responsible for promoting consumer health. In this context, the incorporation of raw materials rich in bioactive phytochemicals and nutraceutical compounds into the development of functional foods is essential for enhancing their physiological efficacy and health-promoting potential [10], while having a positive influence on the consumers’ well-being [11]. Moreover, bioactive compounds such as polyphenols (in particular, flavonoids) and vitamins are more bioavailable through advanced processing methods [2].

Advanced food processing technologies play a critical role in maintaining the structural stability and bioactivity of health-promoting phytochemicals, while concurrently enhancing environmental sustainability through optimized resource efficiency and reduced ecological impact [12].

On the other side, in recent decades, the global incidence of celiac disease and other gluten-related pathologies, such as non-celiac gluten sensitivity and wheat allergy, has demonstrated a significant upward trend. This epidemiological shift has imposed a critical impetus on the food industry to identify and develop alternative gluten-free substrates and formulations, thereby ensuring the nutritional adequacy and technological functionality of products intended for individuals with gluten intolerance [12,13].

From 2018 to 2022, the global market for gluten-free products exhibited an estimated growth of approximately 16%, reflecting a substantial shift in consumer preferences. This trend underscores the emergence of gluten-free formulations as a prominent and enduring driver within the functional and specialized food sectors, ranking among the top ten contemporary developments in food innovation and consumer health-oriented product demand [14].

Recently, Dalal et al., in their 2025 paper [15], emphasized the successful addition of germinated buckwheat flour and buckwheat starch in the gluten-free noodle manufacturing process, using percentage additions of 70–80% and 20–30%, respectively. In line with this, Sturza et al. [16] manufactured buns with a 20% buckwheat flour addition and a 10% buckwheat sprouts addition, highlighting buckwheat’s positive effects on the nutritional value of the final baked products. Moreover, buckwheat flour could be used as a valuable ingredient in protein-rich snack manufacturing, according to Dalal et al., 2025 [15].

Beyond its application in food product development, buckwheat has recently attracted growing interest in the field of food packaging, primarily due to its high starch content. Starch, one of the most abundant natural polysaccharides, has attracted considerable interest in the food packaging sector due to its non-toxicity, biocompatibility, biodegradability, and inherent antimicrobial potential [17].

Therefore, the present review provides a comprehensive analysis of the chemical composition of buckwheat, emphasizing its nutritional and bioactive properties and their implications for human health. Additionally, it explores emerging applications of starch buckwheat, particularly its potential utilization in the biodegradable packaging industry.

### 1.1. Brief History and Buckwheat Varieties

From the 16th century onward, during the Spanish colonization of South America, native foods and traditional practices were gradually replaced by those influenced by European culture [18]. As part of this exchange, new cereal crops such as barley (*Hordeum vulgare* L.), buckwheat (*F. esculentum* Moench), and wheat (*Triticum turgidum* L.) were introduced and integrated into local food systems [18]. At the beginning, these grains were first meant for domestic use, but as time went on, they became vital staples for low-income households [19].

Pseudocereals are plants that, although not belonging to the grass family, produce fruits and seeds that can be used to manufacture staple foods like bread. Pseudocereals are dicotyledonous plant species, in contrast to conventional cereals such as wheat, rice, and barley, which are monocotyledonous. However, due to the comparable nutritional composition and functional properties of their seeds to those of cereals, they are classified as ‘pseudocereals’ [20]. Based on these considerations, buckwheat is a pseudocereal with particular interest due to its agronomic, nutritional, and functional attributes.

Buckwheat is scientifically known as *F. esculentum* Moench, and it and quinoa (*Chenopodium quinoa* Willd) are the most widely cultivated and commercially significant pseudocereal crops worldwide [18]. Following rice, buckwheat holds the position of the second most significant grain crop in Japan in terms of cultivation and consumption [20]. Southwestern China is believed to be the primary domestication region of buckwheat [21], but also its place of origin [22]. By the thirteenth century, buckwheat cultivation had been established in several parts of Europe, including Italy, Austria, and Germany [20]. Today, it is widely cultivated in numerous countries where grain crops represent a significant component of agricultural systems [22]. Regarding buckwheat cultivation requirements, buckwheat exhibits optimal growth under warm climatic conditions and demonstrates adaptability to a wide range of soil types, except for sandy soils, which are generally unsuitable for its cultivation [23].

The *Fagopyrum* genus comprises fewer than 30 species, which are mostly endemic to southern China [24]. The most widespread species of the genus *Fagopyrum* are common buckwheat (*F. esculentum* Moench) and Tartary buckwheat (*F. tataricum* (L.) Gaertn) [24]. According to statistics, the most widespread species of *F. esculentum* is distributed in Asia, Europe, Africa, Oceania, and North America. In Italy, it thrives in the alpine regions of Valtellina and Val Venosta, where it is used for the preparation of typical regional food products [25].

Compared to common buckwheat, Tartary buckwheat contributes to biodiversity and sustainable agriculture. Originating from western China, Tartary buckwheat adapts better to harsh climate conditions than common buckwheat. Tartary buckwheat is mainly grown in the mountainous areas of southwestern China [25]. In Europe, Tartary buckwheat is grown in Luxembourg, Belgium, Germany, Slovenia, Italy, Ukraine, and Serbia [25].

One of the basic characteristics of Tartary buckwheat is its resistance to cold and drought, as well as resistance to UV-B radiation. This characteristic is associated with its high content of polyphenols, rutin, quercetin, and fagopyrin, which have a protective role [26]. Researchers have shown that buckwheat has a flavonoid content 25–50 times higher than wheat and corn [27]. The presence of a high content of polyphenols in the composition of tartaric buckwheat has a role in protecting the plant against unfavorable climatic conditions, plant diseases, and pests [28].

Tartary buckwheat (*F. tataricum*) is considered a nutritionally valuable ingredient due to its high concentrations of dietary fiber, high-quality proteins, essential amino acids, vitamins, and minerals. Buckwheat crops are recognized for their high protein content [23]; it is a versatile grain that is highly valued in Japan for its health benefits, including diabetes prevention and cholesterol reduction [23].

Owing to its comprehensive nutritional profile and documented health-promoting properties, buckwheat serves as a promising raw material for the formulation of novel functional food products such as bread, biscuits, and noodles [29].

### 1.2. Cultivation and Production

**Figure 1:**
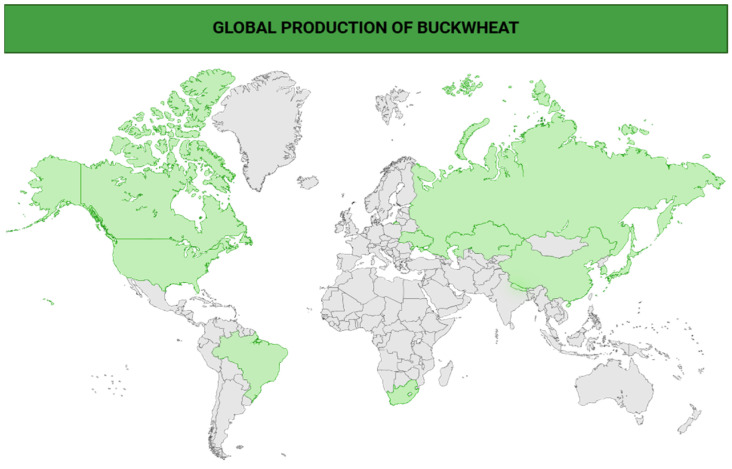
Buckwheat global production in 2022, adapted and modified from [30,31].

From the middle of the 6th millennium BC, buckwheat came from China [21]. In the 3rd millennium BC, it also spread in Europe via trade routes that connected the Himalayas to the Caucasus and Europe [21]. Figure 1 highlights an overview of worldwide buckwheat production.

In 2022, Russia led global buckwheat production with 1,222,382 tons, followed by China and Ukraine [30]. According to data from the Food and Agriculture Organization (FAO), in 2022, buckwheat production was 2,231,443 tons. Compared to 2021, buckwheat production increased by 16.3%. According to history, buckwheat production reached its peak in 1992 with a world production of 4,975,23 tons. On the other hand, the lowest production was recorded in 2010, amounting to 1,454,590 tons. Since 1961, the average annual growth rate has been −0.170%. Russia accounted for 54.7% of global buckwheat production in 2022 [30].

The harvest area and production of buckwheat stayed relatively high until 2002, but production has steadily declined since then, reaching its lowest point in 2017—a drop of almost 3,036,090.56 tons [31]. Two species are cultivated globally: common buckwheat (*F. esculentum* Moench) and Tartary buckwheat (*F. tataricum* L.). Common buckwheat is produced in Asia, Europe, and North America. In comparison with Tartary buckwheat (*F. tataricum* L.), common buckwheat has a much larger planting area and yield [10,31].

The increasing variability in recent years, driven by extreme weather conditions and fluctuations in seed prices, will make long-term planning increasingly essential. Buckwheat shows broad adaptability, with temperate and subtropical climates being particularly favorable for its cultivation due to the typically adequate and well-distributed rainfall throughout the growing season [32].

Therefore, in Japan, after its domestication and widespread adoption, buckwheat (*F. esculentum* Moench) has acquired substantial agronomic relevance, being classified as a strategically important grain crop due to its adaptability, nutritional value, and role in traditional food systems [20].

Buckwheat crops are both environmentally and economically sustainable. It requires minimal agricultural inputs, such as chemical and synthetic fertilizers and fossil energy [33]. This advantage contributes to the preservation of global ecosystems and habitats while also addressing freshwater scarcity [33]. Due to its minimal watering needs, buckwheat farming encourages more environmentally friendly resource management [33,34].

The wide distribution of buckwheat across the globe is mainly due to the following reasons: a short growth period, tolerance to poor soil conditions, drought resistance, and strong adaptability [34]. In 2020, China was the greatest producer of buckwheat, with a cultivation area of 6.25 × 10^5^ ha^−1^ and a production of 5.04 × 10^5^ tons [35]. Buckwheat production exhibits a low yield per hectare, due to limiting factors. Weed infestation is the primary constraint to the intensive cultivation of buckwheat [36].

It has been suggested that sustainable intensification is a workable way to meet the nutritional needs of an expanding population while reducing resource inputs and concurrently preserving or improving natural ecosystem services [37].

Buckwheat was first grown traditionally, particularly in regions with a temperate climate. Currently, *F. esculentum* Moench is cultivated across diverse agroecological zones worldwide and exhibits optimal growth and adaptability under Mediterranean climatic conditions, which are typified by moderate, precipitation-rich winters and arid, high-temperature summers. Maximum yields are obtained when flowering takes place between 18 and 23 °C, while the ideal germination temperature for buckwheat is roughly 10 °C [38].

Buckwheat cultivation is influenced by agroclimatic conditions and temperature [38].

It is considered a short-lived crop that usually grows in a cool and wet climate. It is grown in well-drained sandy soils but can also grow in acidic soils with a pH < 5 [39]. Preparing the land for growing buckwheat does not require extensive work, because buckwheat has the ability to grow even in very poorly worked soil [39]. Prior to its cultivation, it is recommended to implement pre-sowing land preparation several weeks in advance. This agronomic practice facilitates effective weed suppression and enhances soil structure and porosity, thereby promoting optimal seedbed conditions for germination and early seedling development [40]. Soil drainage must be done in such a way as to avoid the seeds from sinking, because water contact with seeds will affect the germination process and may reduce crop yield [40]. The right sowing date must be chosen in order to take advantage of all the plant’s beneficial qualities. The goal of sowing preparation is to determine the best time for different plant varieties such that environmental conditions support plant germination and survival [39].

According to Lee et al. [41], the best sowing date for buckwheat in China is likely to be late May, resulting in a maximum production yield of 2.059 kg/ha, compared to late April and late June in China [38]. According to several studies, in Western Europe, it has been found that the optimal sowing date is between May and July, because the risk of frost at the end of summer is minimal [38]. The growing season varies depending on location and susceptibility to frost. Also, sowing differs depending on altitude. At low altitudes, it is cultivated between May and August, and at high altitudes, it is cultivated between April and May [42]. During cultivation, it is advised to sow buckwheat seeds at a depth of 4–6 cm to ensure adequate soil coverage, promote uniform germination, and support early seedling establishment under field conditions. If the climatic conditions are dry, the seeds need higher humidity to germinate, so they are sown at a depth much greater than 6 cm [38]. In a normal way, buckwheat is sown in rows, keeping a distance of 10 cm between rows, or it can be spread randomly across the field. The seeding rate ranges from 35 to 50 kg/ha when cultivated as a grain crop. To avoid low yield, the seeding rate must be controlled as excessive density can have a negative impact on plant growth [38].

The yield of buckwheat is mainly influenced by soil nutrients, which can be analyzed through soil tests. Although it is not regarded as a strong nitrogen consumer, buckwheat responds better to a balanced fertilizer program. Because of its quick growth and biomass output, buckwheat is regarded as an effective crop that can enhance the quality of soil nutrients, particularly nitrogen. According to a field study, the soil needs to be fertilized in the following ways in order to produce the maximum yield when producing buckwheat: each hectare contains 40 kg of potassium, 22 kg of phosphorus, and 47 kg of nitrogen [38].

Buckwheat requires more potassium in the soil if it is planted in sandy or clayey soils. Additionally, buckwheat exhibits lodging and produces lower yields in fields with high nitrogen contents; however, this effect is mitigated by a higher phosphorus dosage. After the buckwheat seeds have been sown, and when the climatic conditions have been favorable, harvesting can be done 10 weeks after planting [38]. Harvesting begins when 70–75% of the seeds have reached physiological maturity and the plants have lost most of their leaves. At this stage, the lower seed heads begin to shatter. After harvesting, buckwheat needs to be dried, and the optimal conditions for carrying out this stage require a temperature of 45 °C and a humidity of 16%. It is not recommended that buckwheat be stored for a long period of time, as it is prone to rancidity [38].

### 1.3. Buckwheat Sustainability

The impacts of global warming, including increasingly erratic climatic events, shifting precipitation patterns, and reductions in arable land availability, pose significant challenges to global agriculture [43]. In response, researchers are actively exploring strategies to enhance the resilience of agroecosystems through the implementation of sustainable agronomic practices [43]. Among the crops of growing interest is buckwheat (*Fagopyrum* spp.), which has attracted considerable attention from the agro-food industry due to its notable nutritional value and suitability for gluten-free food products [44,45].

Buckwheat is particularly recognized for its high adaptability to diverse environmental conditions, a characteristic that positions it as a promising climate-resilient crop. It demonstrates tolerance to a variety of abiotic stressors, including drought, elevated temperatures, cold stress, ultraviolet radiation, heavy metal contamination, and various plant pathogens [46]. One of the physiological attributes contributing to its resilience is its complex root system, which enables efficient water uptake from deeper soil layers, thereby supporting plant survival during drought conditions. Furthermore, buckwheat possesses a deep and fibrous root system that contributes to the improvement of soil structure by enhancing porosity and facilitating the infiltration of water and air. Additionally, its rapid shoot development provides effective ground cover, thereby reducing the risk of wind and water erosion while further promoting water infiltration. This vegetative growth also contributes to soil fertility through the addition of organic matter, as its biomass decomposes and enriches the soil [47].

Recent studies have also indicated that buckwheat’s tolerance to extreme temperatures is modulated by epigenetic mechanisms, suggesting a potential for enhanced adaptability through molecular and genetic responses to environmental stimuli. This adaptability allows buckwheat cultivation across a broad spectrum of climatic zones, ranging from temperate to subtropical and high-altitude regions, provided that conditions remain free from prolonged frost or snow cover [48].

However, while buckwheat is relatively tolerant of a range of environmental conditions, it is susceptible to stress in excessively cold and water-saturated environments, which can compromise germination and yield [46]. Optimal germination occurs at temperatures no lower than 7 °C, with ideal vegetative growth observed between 16 °C and 22 °C. Depending on the geographic region, buckwheat can be cultivated within a broader thermal window ranging from 10 °C to 30 °C [38].

In terms of edaphic requirements, buckwheat is well-suited to a wide variety of soils, excluding poorly drained, heavy clay, and excessively sandy substrates. It performs optimally on well-drained, moderately fertile soils and is notably productive even on marginal and low-input lands. Interestingly, excessively fertile soils may lead to reduced grain yield, likely due to imbalanced vegetative growth [38]. Pre-sowing soil preparation—including weed control and soil loosening via one to two rounds of plowing—has been shown to enhance seed germination and early plant establishment [49].

Regarding fertilization, empirical studies indicate that buckwheat responds more favorably to organic fertilizers than to synthetic chemical inputs, further underscoring its compatibility with sustainable and low-input farming systems [38].

In summary, buckwheat can be characterized as a sustainable and resilient crop, owing to its low input requirements, broad agroecological adaptability, and compatibility with organic farming practices. These attributes make it a suitable candidate for integration into climate-smart agricultural systems aimed at ensuring food security and environmental sustainability.

## 2. Chemical Composition

In the last decades, consumers have been concerned about their health and their food diet. Buckwheat is a pseudocereal that is used as food, valued for its beneficial properties and health advantages. Moreover, buckwheat is a grain with an excellent nutrient profile which comes to the aid of people suffering from celiac disease and malnutrition [50].

Compared to staple crops such as wheat, rice, and quinoa, buckwheat is considered superior in terms of its nutritional profile, owing to its higher concentrations of bioactive compounds and essential nutrients [19]. Therefore, buckwheat is used in various pharmaceutical treatments [51]. Table 1 displays the proximate composition of the main buckwheat species.

### 2.1. Carbohydrates

The buckwheat seed comprises three primary anatomical components: the hull, the endosperm, which is rich in starch and proteins, and the germ, which, along with the bran, encompasses the outer layers of the seed [56]. Starch is the main source of carbohydrates in buckwheat seed and varies between 58 and 73% [50,55].

After processing the buckwheat seeds, flour is obtained. The obtained flour also contains starch. The starch content varies between 39 and 43% and is dependent on several factors, for example, the buckwheat variety and milling process [55]. For instance, the wet milling process led to a significant reduction in the damaged starch content of the resulting flour. This effect can likely be attributed to the high water usage inherent to the wet milling of soaked common buckwheat grains. During milling, the presence of water molecules may function as plasticizers, enhancing the elasticity of starch granules. This increased flexibility likely improves the granules’ fracture toughness, thereby reducing their susceptibility to mechanical damage during processing [57]. This idea is also supported by [56], whose authors demonstrated that the type of milling has a direct influence on the damage degree of starch granules and on its content, highlighting that wet milling obtained the best results compared with roller milling, ultrafine milling, and stone milling processes. On the other side, the extent of starch damage during milling is influenced by several processing parameters, including milling intensity, rotor speed, the type of milling equipment employed (e.g., impact mills or roller mills), milling duration, thermal buildup during the process, and the specific pre-treatment or conditioning techniques applied to the raw buckwheat material prior to milling [58].

Factors such as cultivar or strain, climatic and edaphic conditions, as well as post-harvest storage practices influence the chemical content of buckwheat [59].

Buckwheat starch is primarily composed of two polysaccharide fractions: amylose and amylopectin, which typically constitute approximately 25% and 75% of its structure, respectively [50]. On the other side, Jha et al. [55] mentioned values of approximately 39% and 43% for buckwheat amylose content in Tartary buckwheat and common buckwheat.

Buckwheat starch granules are characterized by their small size (ranging from 3 to 10 μm), smooth surface morphology, and predominantly polygonal shape, exhibiting structural similarities to both tuber and cereal-derived starches [50].

Thermic processes such as autoclaving and boiling affect the content of resistant starch in buckwheat. At elevated temperatures, changes occur in starch structure, including starch gelatinization, alterations in the cellulosic composition, and degradation of non-covalent interactions [60].

Buckwheat-derived products are recognized for their elevated levels of resistant starch, a functional carbohydrate associated with numerous health-promoting effects. These include a reduced risk of colorectal cancer, hemorrhoids, diverticulosis, and constipation, along with enhanced fecal bulk and improved regulation of glycemic response and blood lipid profiles. Additionally, resistant starch has been reported to exhibit prebiotic properties, contributing to beneficial modulation of the gut microbiota [61]. Furthermore, it has been reported that polyphenols and lipids present in buckwheat flour can form non-covalent complexes with starch, which can inhibit α-amylase activity. This interaction may contribute to a reduction in starch digestibility, thereby exerting a potentially beneficial effect in the dietary management of hyperglycemia [55].

### 2.2. Proteins

Pseudocereals are an important source of protein. Moreover, buckwheat is a significant source of protein, which can vary from 8.5 to 19%, being dependent mainly on the cultivar [59]. When compared to major cereal crops such as wheat (*Triticum* spp.), maize (*Zea mays* L.), and rice (*Oryza sativa* L.), buckwheat exhibits a relatively higher protein content, typically ranging between 10% and 15%, thereby highlighting its superior nutritional profile in terms of protein concentration [53]. According to the findings reported by Taylor et al. [62], the buckwheat protein fractionation profile reveals that globulins represent approximately 43.3–64.5% of the total protein content, followed by albumins (12.5–18.2%), glutelins (8.0–22.7%), prolamins (0.8–2.9%), and residual proteins, accounting for approximately 15%. Proteins that are associated with celiac diseases (30 kDa *prolamins*) are absent in buckwheat.

Among the buckwheat proteins, 2S albumins and 8S and 13S globulins have a similar structure to the legumin storage proteins. The globulin proteins found in buckwheat have a hexameric structure consisting of acidic (32–43 kDa) and basic (23–25 kDa) polypeptide subunits connected by disulfide bonds [50].

It is worth noting that buckwheat proteins have a well-balanced composition of amino acids [63] and that buckwheat protein possesses a high biological value, amounting to 92.3% of that of egg protein [59]. Buckwheat amino acids are characterized by a high lysine content and exhibit a distinct amino acid profile compared to conventional cereal proteins, with lower levels of glutamic acid and proline, and elevated concentrations of arginine and aspartic acid [63].

### 2.3. Dietary Fibers

The research conducted in the field identifies pseudocereals as the most effective source of dietary fiber. In this context, buckwheat is recognized for its abundant content of dietary fiber [64]. The dietary fiber content in buckwheat differs based on the level of processing—23.8% in unhusked seeds, 10.3% in husked seeds, and 7% in groats—and also varies among different cultivars [50]. Studies have demonstrated that the milling process adversely affects dietary fiber content on the buckwheat seed coat [65]. For instance, buckwheat bran, a byproduct obtained during the buckwheat milling process, is made up of a percentage of 40%dietary fiber [65]. On the other side [66], it should be highlighted that the sprouting process significantly enhanced the crude fiber content of buckwheat, with levels increasing from 7.80% to 9.74%, indicating improved dietary fiber accumulation during germination.

Dietary fibers are divided into two categories: soluble fibers, such as pectin and gums, and insoluble fibers, including lignin and cellulose. Buckwheat has been shown to exert beneficial health effects, particularly in lowering serum cholesterol levels and mitigating obesity. These physiological effects are largely attributed to its dietary fiber content, comprising approximately 4.8% soluble fiber and 2.2% insoluble fiber [50]. The dietary fiber content of buckwheat represents a critical component of its nutritional and functional profile, particularly in relation to gastrointestinal and metabolic health. Buckwheat contains dietary fiber, which functions synergistically to enhance intestinal motility, attenuate postprandial glycemic responses, and reduce serum cholesterol concentrations [67].

Given its naturally high fiber content, especially in whole or minimally processed forms, buckwheat is well-suited for incorporation into gluten-free diets and the formulation of functional food products with targeted health benefits [68]. Dietary fiber plays a fundamental role in human nutrition by supporting gastrointestinal health, modulating blood glucose levels, and lowering serum cholesterol concentrations, which collectively help reduce the risk of cardiovascular disease and type 2 diabetes [68]. Soluble fiber slows gastric emptying and nutrient absorption, thereby enhancing satiety and contributing to effective weight management. In contrast, insoluble fiber promotes bowel regularity and supports a healthy gut microbiota. Consistent intake of fiber-rich foods is strongly associated with a decreased incidence of chronic diseases and improved metabolic outcomes [68].

On the other side, it is important to mention that soluble dietary fiber exhibited superior functional properties, including enhanced water-holding capacity, oil-holding capacity, and cholesterol and glucose adsorption capacity. Additionally, soluble dietary fiber demonstrated greater antioxidant activity and a higher capacity for gel formation [65].

### 2.4. Lipids

According to the chemical composition (Table 1), the lipids have a low value in buckwheat, ranging from 1.5% to 4.7%. In buckwheat grains, retrieved lipids are divided into neutral lipids, at 81–85%, phospholipids, at 8–11%, and glycolipids, at 3–5%. The lipid profile of buckwheat grains consists predominantly of neutral lipids (81–85%), with phospholipids and glycolipids comprising approximately 8–11% and 3–5%, respectively [50]. Due to the high content of fatty acids, buckwheat is recognized for its benefits against heart diseases, cancer, and diabetes. Even if the fatty acid content is low, it plays a crucial role in determining the quality of food [69]. The fatty acids present in buckwheat include palmitic, stearic, oleic, linoleic, linolenic, and eicosanoid acids [50]. Both common buckwheat (*F. esculentum* Moench) and Tartary buckwheat (*F. tataricum* L.) have approximately the same content of fatty acids, 4.4%. The fatty acid composition of buckwheat is characterized by a high proportion of unsaturated fatty acids, which are beneficial for human health [50].

Studies have shown that unsaturated fatty acids constitute approximately 78.7% to 82% of the total fat content in various buckwheat flours [70]. The main saturated fatty acid identified in buckwheat is palmitic acid (C16:0), while linoleic and oleic acids are the main fatty acids identified by Sinkovic et al. [71]. The ω-6/ω-3 ratio is 1.79 for conventional buckwheat, while organic buckwheat reached an amount of 1.75, showing that buckwheat could be a source of balanced intake of ω-6 and ω-3 fatty acids [72]. This ratio is highly important, mainly in preventing the progression of obesity, especially among children and adolescents, who represent a high-risk population group [72].

Notably, processing methods such as germination and extrusion do not significantly alter the fatty acid composition of buckwheat flours, but, on the other hand, the production season, genetic background, cultivation practices, and milling process significantly affect the fatty acid composition of buckwheat [71]. For instance, the highest amount of saturated fatty acid was identified in the buckwheat hulls, while bran exhibited the lowest amount [71].

This favorable fatty acid profile, with a high unsaturated to saturated fatty acid ratio, underscores the nutritional value of buckwheat and its potential role in promoting cardiovascular health and reducing the risk of chronic diseases [3].

### 2.5. Vitamins and Minerals

Vitamins represent a group of organic compounds that are essential for the normal functioning of the human body and required in small amounts [73]. Choline, followed by tocopherol and niacin, are the main vitamins identified in buckwheat grains, with values of 440 mg/100 g, 40 mg/100 g, and 18 mg/100 g, respectively [52]. Buckwheat is rich in niacinamide, vitamin K, and choline. Moreover, buckwheat contains carotenoids, such as lutein and zeaxanthin [52]. Tartary buckwheat has an abundance of B group vitamins in comparison with common buckwheat. Also, common buckwheat is richer in vitamin E compared to Tartary buckwheat. The common buckwheat is deficient in minerals such as Co, Fe, Ni, and Se in comparison with Tartary buckwheat [4].

Macroelements are present in moderate amounts, such as P, K, Mg, Ca, Fe, and Zn. Microelements are mainly concentrated in the covering, shell, and aleurone layers of the seeds [74]. The majority of mineral elements such as magnesium (Mg), manganese (Mn), iron (Fe), copper (Cu), and zinc (Zn) are predominantly complexed with phytate (myo-inositol hexakisphosphate), a form that significantly impairs their bioavailability due to the strong chelation and poor solubility of these phytate–mineral complexes under physiological conditions [75]. It is also important to outline that bioaccessibility refers to the proportion of bioactive compounds that are liberated from the food matrix during gastrointestinal digestion, rendering them available for absorption in the small intestine. In contrast, bioavailability denotes the fraction of ingested bioactive constituents that successfully reach the systemic circulation and are subsequently distributed to target organs and tissues, where they exert their physiological effects [76].

Minerals such as Zn, Co, and K become bioavailable for absorption following enzymatic digestion, which converts them into a soluble form [73]. In line with this, Klepacka et al. [73] demonstrated that buckwheat husk removal exerted a markedly negative impact on both the total manganese content and its bioaccessible fraction as determined by enzymatic digestion. In contrast, husk removal had a beneficial effect on zinc, enhancing its post-digestion concentration by approximately 50%. Furthermore, it was emphasized that short-term thermal treatment of buckwheat at lower temperatures can enhance the bioaccessibility of minerals, with a particularly pronounced positive effect observed for manganese and magnesium [73]. Fermentation, germination, and soaking are considered important tools that might increase the mineral amount and their bioavailability [75].

Gluten-free products, often characterized by their limited content of vitamins and minerals, can benefit from the inclusion of buckwheat, which significantly increases the concentration of essential vitamins and minerals in the diet [20].

Vitamins and minerals are indispensable micronutrients required for numerous biochemical and physiological processes essential to human health [3]. The B-complex vitamins play a central role in energy production, neurological function, and the synthesis of red blood cells [3]. Vitamin K is primarily involved in the activation of clotting factors necessary for blood coagulation and also contributes to bone health by regulating calcium deposition. Deficiencies in these micronutrients can lead to a range of adverse health outcomes, including anemia, cognitive dysfunction, impaired hemostasis, and decreased bone mineral density [3].

## 3. Bioactive Compounds, Health Effects, and Thermostability

Over the past decade, there has been a marked increase in interest among European consumers regarding health-promoting and nutritionally balanced food products. According to a large body of literature, colon cancer is a cause of death, and in 2012, 694,000 deaths were recorded globally. The likelihood of developing this disease increases with age, but its incidence is significantly lower among vegetarians and those who avoid meat and dairy products [77].

The content of flavonoids, phenolic acids, amino acids, and thiamine in buckwheat has been highly investigated. Rutin is a plant-derived secondary metabolite known for its potent antioxidant properties [78]. Common, Tartary, and golden buckwheat varieties are notable for their high rutin content, which is present in various edible parts of the plant, including both leaves and seeds [79].

The concentration of rutin in buckwheat seeds has been reported to range from 6 to 38.9 mg/g, relative to body weight [10]. Research on buckwheat seeds (*Fagopyrum* species) has focused on the isolation of the flavonoid rutin and the investigation of its potential anticancer activity against osteosarcoma cell lines (SAOS-2) [80]. The findings of the present study demonstrate that the rutin fraction exerts an inhibitory effect on the proliferation of SAOS-2 osteosarcoma cells in a time- and dose-dependent manner and therefore induces osteosarcoma cell apoptosis [80].

In response to this interest, researchers set out to evaluate the impact of in vitro digested buckwheat bran on the inhibition of HT-29 cancer cell proliferation. The results revealed that digested buckwheat bran contains high levels of bioactive compounds, including rutin, quercetin, catechin, and peptides rich in serine, proline, glycine, histidine, and arginine, which exhibited a strong cytotoxic effect on colon cancer cells, likely due to synergistic interactions among these compounds. These findings highlight the potential of buckwheat byproducts as functional ingredients in pro-health food products, though further molecular studies are needed to confirm the mechanisms involved [77].

In this line, free phenolic extract from Tartary buckwheat bran demonstrated anticancer activity against human breast cancer MDA-MB-231 cells in a dose-dependent manner. This inhibitory effect was mediated via activation of the p38/MAPK signaling pathway, as evidenced by the upregulation of phosphorylated p38 (p-p38) and phosphorylated ASK1 (p-ASK1), along with the downregulation of TRAF2 and phosphorylated p53 (p-p53), leading to the induction of apoptosis. Additionally, the extract suppressed cell cycle progression from the G1 to S phase by increasing the expression of the cyclin-dependent kinase inhibitor p21 and concurrently reducing the expression of proliferating cell nuclear antigen (PCNA), cyclin D1, and CDK4 [81].

Human gastric carcinoma cell line MGC80-3 was utilized to evaluate the antitumor potential of total flavonoid extracts derived from Tartary buckwheat. Among the tested extracts, Tartary buckwheat on the third day of electric field treatment exhibited the most pronounced antitumor activity. This enhanced inhibitory effect is likely attributable to the optimized flavonoid composition achieved following electric field treatment, which may have facilitated a favorable ratio of bioactive constituents responsible for the observed cytotoxic effects [82].

It is important to mention that buckwheat groats are widely used and may contain high levels of bioactive compounds and retrograded starch, which can be beneficial for the prevention of colon cancer and breast cancer [77]. The bioactive compounds found in pseudocereals have a positive impact on human health, by contributing to the prevention of chronic diseases, cardiovascular diseases, and cancer [83].

On the other hand, dietary supplementation with buckwheat flour has demonstrated immunoprotective effects in mouse models exhibiting premature senescence. Notably, multiple components of the innate immune response were enhanced, including macrophage chemotaxis, phagocytic capacity, microbicidal activity, and natural killer (NK) cell activity. Additionally, key aspects of the adaptive immune response showed improvement, such as lymphoproliferative responses to concanavalin A and lipopolysaccharide stimulation, along with increased interleukin-2 (IL-2) secretion [84].

Both in vivo and in vitro investigations suggest that buckwheat possesses gastrointestinal benefits, attributed to its antioxidant and anti-inflammatory properties. However, clinical evidence in humans remains limited, and its definitive effects on human health require further elucidation through well-designed future clinical trials [85].

Multiple studies have demonstrated that buckwheat is widely incorporated into various food products such as bread, noodles, and biscuits. Findings indicate that noodles and biscuits made with buckwheat flour contain rutin concentrations of approximately 1.269 mg/g and 1.421 mg/g, respectively, suggesting their potential contribution to the dietary intake of bioactive compounds [86]. Nonetheless, in baking products made entirely from buckwheat flour, the rutin content was found to decrease to approximately 0.47 mg/g. Interestingly, a significant increase in quercetin content was observed, reaching 4.83 mg/g, likely due to the thermal degradation of rutin into quercetin during the baking process [87]. The degradation and loss of rutin during food processing is well-documented, highlighting the importance of minimizing processing steps in order to preserve its bioactive properties and maximize the health benefits of buckwheat-based products.

On the other side, food processing treatments such as extrusion, microwave, steam explosion, ultrasound, and even superheated steam significantly increased the soluble dietary fiber buckwheat bran content, with a positive impact on molecular size and bound phenolic and monosaccharide composition [65].

Moreover, hydroxycinnamic acids, which are not detectable in native buckwheat flour, become quantifiable following pasta processing. Among the evaluated extrusion parameters, a screw speed of 60 rpm and a raw material moisture content of 30% constituted the optimal conditions for the effective release of trans-*p*-coumaric acid, cis-*p*-coumaric acid, and cis-ferulic acid from their conjugated forms, likely through the disruption of ester and ether linkages with cell wall polymers and other macromolecular complexes [88]. However. Paucar et al. [89] mentioned that thermoplastic extrusion modifies antinutritional factors by denaturing proteins through thermomechanical processing and depolymerizing phenolic compounds—such as condensed tannins—that typically form complexes with proteins. Furthermore, the inherent sensitivity of phenolic compounds to thermoplastic extrusion is attributed not only to the presence of aromatic rings, which are prone to degradation under high thermal and mechanical stress, but also to the occurrence of highly reactive functional groups, such as hydroxyl groups, which further compromise their stability during processing.

In this line, Yalcin [90] showed that the levels of gallic acid, ferulic acid, chlorogenic acid, caffeic acid, rutin, and ellagic acid of gluten-free buckwheat noodles increased with the increase in their buckwheat addition, highlighting the best value at a 60% buckwheat flour addition.

**Figure 2:**
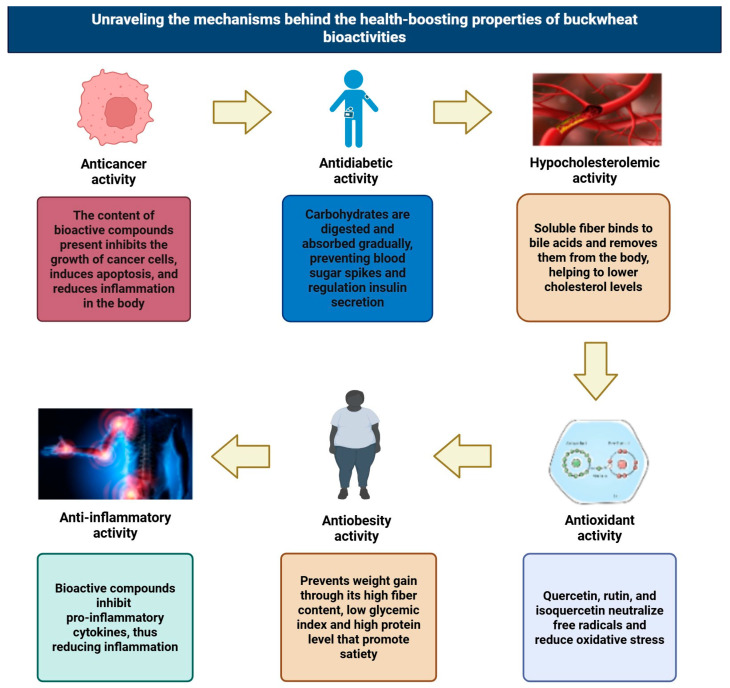
Unraveling the mechanisms behind the health-boosting properties of buckwheat’s bioactive compounds [12,63,83,99].

A more comprehensive summary of the principal bioactive constituents identified in buckwheat, along with their associated health-promoting effects, analytical detection methodologies, and the effective concentration ranges required to elicit physiological responses is displayed in Table 2, while Figure 2 illustrates the multifaceted physiological effects of key bioactive constituents found in buckwheat, including quercetin, rutin, orientin, dietary fiber, and phenolic acids, and highlights six principal health-promoting activities: anticancer, antidiabetic, hypocholesterolemic, antioxidant, anti-inflammatory, and antiobesity effects.

Despite the breadth of scientific evidence underscoring buckwheat’s nutritional and bioactive potential, the current body of literature presents certain limitations. Most studies to date remain at the in vitro or animal model level, with limited large-scale, randomized human clinical trials validating the health benefits of buckwheat’s bioactive compounds, particularly rutin and resistant starch. Moreover, significant variability exists in compositional data due to genotype–environment interactions, processing methods, and analytical protocols, thereby limiting the comparability and reproducibility of results.

**Table 2:** The main bioactive compounds identified in buckwheat, its potential health benefits, and the detection method and concentration needed to exhibit health effects.

Category	Compounds	Detection Methods	Potential Health Benefits	Concentration	References
				Components	
Flavonoids	Rutin	HPLC	Antioxidant Anti-inflammatory Cardiovascular and neuroprotective properties; antibacterial Prevention of human hepatic cancer	1–2 mg/g in leaves, flower, bran	[4,91,92]
	Quercetin			0.02–0.30 mg/g in leaves, flower, seeds, flour	
	Isoquercetin			0.50–1.50 mg/g in leaves, flower, bran, flour	
Anthocyanins	Peonidin, petunidin 3-*O*-glucoside, cyanidin, cyanidin-3-*O*-glucoside, cyanidin-*O*-syringic acid, cyanidin-3-*O*-glucosyl-malonylglucoside	UPLC-ESI-MS/MS	Antioxidant Prevent colon, breast, and prostate cancer and cardiovascular and neurodegenerative diseases Diabetes prevention	1–3 mg/g in leaves, flowers, shell, brain	[93]
Phenolic acids	Gallic acid	RP-UHPLC-ESI/MS	Cardioprotective Antioxidant effects Anti-inflammatory properties Cancer prevention Brain health and neuroprotection Blood sugar control Diabetes prevention	0.10–0.40 mg/g in bran, seeds	[92,94]
	Ferulic acid			0.05–0.15 mg/g in bran and shell	
	4-Hydrobenzoic acid			0.05–0.20 mg/g in bran	
	Isovanilic acid			0.02–0.1 mg/g in bran, whole grains	
	Caffeic acid			0.10–0.50 mg/g in leaves, flowers, and bran	
	Chlorogenic acid			0.10–0.50 mg/g in seeds	
Fagopyrins	Fagopyrin A-F	NMRS-MS	Reduce risk of cardiovascular disease Anticancer properties	3–5 mg/g in leaves, flower, stem	[4,95]
Fagopyritol Steroids	Fagopyritol A_1_ and B_1_	GC-MS NMRS	Reduce risk of cardiovascular disease Anticancer properties	1.50–6.0 mg/g in bran, peeled buckwheat	[95]
	β-sitosterol, β-sitosterol palmitate, daucosterol, ergosterol peroxide, stigmsat-4-en 3,6-dione, stigmast-5-en-3-ol			0.60–1.0 mg/g in bran, whole seeds, peeled buckwheat	
Triterpenoids Phenylpropanoid glycosides	Glutinone, glutinol, olean-12-en-3-ol and urs-12-an-3-ol	HPLC-PDA/LTQ-FTICR-MS	Anti-inflammatory properties Reduce risk of cardiovascular diseases.	0.20–0.70 mg/g in bran, whole seeds	[10,51]
	Diboside A and tatarisides A-G			0.50–1.50 mg/g in leaves, flowers, bran, whole seeds	
Proteins Carbohydrates	Amino acid compositions	HPLC IRS GC NMRS	Better muscle growth and repair Blood sugar control Digestive health Antitumor Hypolipidemic Antidiabetic	14–18% in bran, whole seeds, peeled buckwheat	[93,96]
	Polysaccharides and monosaccharides			70–80% in whole seeds 72–75% in peeled buckwheat 60–65% in bran 10–20% in leaves and flowers	
Fatty acids	_D_-chiro-inositol	GLC GC	Improve heart health Improve brain function Skin health	120–200 mg/g in bran, shell 90–150 mg/g in whole seeds 50–100 mg/g in peeled buckwheat	[10,34,96,97,98]
	Fatty acid composition			4–6% in bran	
	Free fatty acid composition			1.5–2.5% in bran	
Vitamins	Vitamin B_1_	HPLC	Improve immune function Improve eye health Reduce inflammation Skin health	0.0003–0.0060 mg/g in grains and bran	[97,98]
	Vitamin B_6_				
	Vitamin C			0.0003–0.0025 mg/g in leaves, flowers, bran	
Carotenoids	Lutein				
	β-carotene				

Abbreviations: HPLC—high-performance liquid chromatography; UPLC-ESI-MS/MS—ultra-performance liquid chromatography electrospray ionization–tandem mass spectrometry system; RP-UHPLC-ESI-MS—reverse-phase ultra-performance liquid chromatography electrospray ionization–mass spectrometry; NMRS-MS—nuclear magnetic resonance spectroscopy and mass spectrometry; GC-MS—gas chromatography–mass spectrometry; NMRS—nuclear magnetic resonance spectroscopy; HPLC-PDA.LTQ-FTICR-MS—high-performance liquid chromatography photodiode array detector/linear ion trap Fourier transform ion cyclotron resonance hybrid mass spectrometry; IRS—infrared spectroscopy; GC—gas chromatography; GLC—gas–liquid chromatography.

## 4. Buckwheat Starch—A Complex-Based Film

Petroleum-derived flexible plastics and films constitute the predominant form of packaging materials utilized globally. This widespread adoption is primarily attributed to their superior processing characteristics, effective water vapor barrier properties, high optical clarity, and cost-effectiveness [100]. By posing serious dangers like resource depletion, greenhouse gas emissions, pollution, and the buildup of plastic trash in the oceans, packaging contributes significantly to climate change and global warming [101]. The significant need to address the environmental, financial, and social issues associated with traditional packaging has led to the introduction of sustainable packaging into the market [102]. The increasing demand for environmentally sustainable packaging solutions has directed attention toward biodegradable materials, especially those derived from renewable resources [101].

Natural biopolymers such as proteins, polysaccharides, and lipids are most commonly used in the development of packaging materials. From the polysaccharides, starch exhibits favorable film-forming capabilities, low oxygen permeability, and neutral sensory characteristics, making it a suitable and sustainable material for edible and biodegradable packaging applications [17].

Starch-based edible films have several useful properties that make them special for use in food packaging. Accordingly, the products’ improved nutritional makeup and extended shelf life, and the antimicrobial and antioxidant properties of the films, all contribute to maintaining their freshness and safety [103].

Edible starch films can act as carriers for antioxidants, essential compounds to stop oxidative reactions and neutralize free radicals, and can thus protect food from oxidative degradation and extend its shelf life [104]. According to Cui et al. [104], the addition of antioxidants, including Vitamin C, Vitamin E, flavonoids, phenolic acids, and carotenoids, aids in the removal of reactive oxygen species and stops lipid oxidation, protein breakdown, and color changes in packaged goods [104]. Furthermore, starch films can serve as effective carriers for antimicrobial agents or essential oils, thereby enhancing microbial stability and ensuring food safety [104].

The rising global focus on environmental sustainability has led to an increase in biodegradable films made from natural biopolymers like polysaccharides, proteins, and lipids. Starch-based films stand out as promising eco-friendly alternatives to petroleum-based plastics due to their renewable nature and biodegradability [105,106].

Among polysaccharide-based films, starches from conventional sources like corn, potato, and wheat have been extensively studied. Their mechanical and barrier properties are largely influenced by the amylose-to-amylopectin ratio, molecular structure, and crystallinity pattern. Corn and wheat starches, for example, contain relatively high amylose contents and exhibit B- or A-type crystallinity, respectively, contributing to differences in film properties such as tensile strength, flexibility, and water vapor permeability [102,105].

On the other hand, buckwheat starch has recently emerged as a promising candidate for sustainable packaging applications. With a typical amylose-to-amylopectin ratio of approximately 25:75 and A-type crystallinity, buckwheat starch shares structural similarities with cereal starches but also presents unique advantages [105,106]. Its relatively low molecular weight and slower retrogradation rate improve its stability during the film manufacturing process and storage [107,108]. Buckwheat starch is characterized by a relatively low gelatinization temperature, a favorable attribute that facilitates energy-efficient processing and enhances its suitability for film-forming applications. Despite these advantageous characteristics, its potential application in film production remains largely underexplored in the scientific literature [100]. On the other side, Tartary buckwheat starch also has some deficiencies, such as low viscosity, swelling power, and absorption capacity that could be improved through thermal treatments (heat fluidization), leading to a better morphological structure, starch gelatinization, and A-type starch crystallinity [109]. Ultrasound is another technique that has gained the attention of researchers, and it is defined as a mechanical wave that transmits energy to the sample through a phenomenon known as acoustic cavitation, characterized by the formation, growth, and subsequent collapse of microbubbles within the medium. Ultrasonic buckwheat starch treatment could also improve its physical properties, such as loss modulus and crystallinity, and facilitate its processability, as mentioned by Yan et al., 2025 [110].

In contrast to conventional plastic polymers, biopolymers such as starch-based films exhibit limited applicability as packaging materials due to their inherently poor mechanical strength and inadequate water barrier properties, which render food products more vulnerable to environmental influences [111]. To improve their performance, researchers have explored structural modification and the inclusion of hydrophobic compounds [112]. Techniques such as lipid complexation, crosslinking, and the addition of copolymers have proven effective in enhancing film functionality [113]. Plasticization, the addition of additives, changes in pH, nanoparticles, and chemical crosslinking have also been studied as useful strategies needed to improve starch-based film performance [111].

Buckwheat starch can be modified through various methods—such as the addition of citric acid or tartaric acid, the incorporation of zinc oxide, and high-pressure or heat–moisture treatments—to enhance its mechanical, barrier, and antimicrobial properties [114]. For instance, citric acid, a GRAS (Generaly Recognised As Safe) listed tricarboxylic acid, serves both as a plasticizer and crosslinker by forming covalent bonds between its carboxyl groups and starch hydroxyl groups. This modification improves the films’ mechanical strength, thermal stability, and moisture resistance [111]. Henning et al., 2025 [100] showed that buckwheat starch films with the addition of tartaric acid exhibited significant enhancements in water vapor permeability (WVP) and thermal stability, while maintaining comparable tensile strength and demonstrating improved elongation relative to films without the addition of organic acid. Based on the findings, the incorporation of tartaric acid emerged as the most promising formulation strategy, particularly for applications requiring effective protection against environmental humidity.

Recently, Thakur et al. [17] developed pH-sensitive films via the casting method using buckwheat starch (5% dry basis) as the primary film-forming matrix, citric acid as a crosslinking agent, and natural rose petal extract as a pH-responsive indicator. The final result showed that incorporating 5% citric acid and 6.36% rose petal extract demonstrated the most favorable properties, indicating its potential application as an intelligent packaging material for real-time monitoring of food quality and safety.

Lately, Bansal et al. [115] aimed to develop an innovative antimicrobial film incorporating β-cyclodextrin and curry leaf essential oil encapsulated within a buckwheat starch and chitosan matrix, aiming to improve green grapes’ shelf life. The results showed promising results regarding the functional properties and the film’s hydrophobicity. Additionally, an active packaging film based on buckwheat esterified starch and chitosan was used to prolong the shelf life of mutton meat by 2 days [116].

However, limitations like reduced elasticity, discoloration, and low elongation at break have been observed. Further studies are needed to optimize buckwheat starch-based films for improved performance in packaging applications [105].

Amylose–lipid complexation offers a sustainable method for modifying starch, requiring fewer chemicals and less energy while producing biodegradable materials [117]. These complexes form during starch gelatinization, when amylose interacts with fatty acids through hydrophobic interactions to create single-helical inclusion complexes. As a result, the modified starch exhibits enhanced hydrophobicity and elasticity, making it suitable for advanced packaging applications [118].

Koca et al. [105] have introduced, for the first time, a starch–lipid complex formed from buckwheat starch and capric acid, with the film production process optimized using response surface methodology. The optimized films were thoroughly characterized to evaluate their mechanical, barrier, and structural properties, highlighting their potential for sustainable packaging applications. Mechanical properties were prioritized in optimizing the film-forming process, as they reflect the film’s durability and cohesion. These characteristics are essential for protecting food during processing and transport [118]. Recently, Sarker et al., 2025 [111] showed that methods such as ultrasonic treatment, the addition of chitosan nanoparticles, and thermal treatment could greatly improve tensile strength, lower elongation at break by 70%, and improve resistance to water absorption of buckwheat starch films.

To conclude, we can assess that a key gap lies in the underexploited industrial application of buckwheat starch in biodegradable packaging. While chemical modifications (e.g., citric/tartaric acid crosslinking), incorporation of antimicrobial agents, and physical treatments (e.g., ultrasound, thermal processing) have shown promising results in improving film performance, most studies lack standardization and fail to address scalability, shelf-life behavior, and long-term environmental degradability. Moreover, current research remains largely empirical, with insufficient mechanistic insight into structure–function relationships and limited use of advanced material characterization techniques. From a critical perspective, buckwheat starch films offer theoretical advantages, but their commercial viability is constrained by technological limitations and the absence of comprehensive life cycle or toxicological assessments. Future work should prioritize multi-parameter optimization, interdisciplinary modeling, and validation under real-world packaging conditions.

## 5. Conclusions and Future Perspectives

Buckwheat (*Fagopyrum* spp.), a dicotyledonous pseudocereal from the Polygonaceae family, has re-emerged as a crop of substantial agronomic, nutritional, and functional importance. Its unique phytochemical profile, including high levels of flavonoids (notably rutin and quercetin), phenolic acids, resistant starch, high-quality proteins, essential amino acids, and dietary fiber, confers a wide array of documented health-promoting properties. These include anti-inflammatory, antioxidant, antidiabetic, hypocholesterolemic, and anticancer effects. In addition, its naturally gluten-free nature positions buckwheat as a valuable ingredient in the development of functional food products tailored for individuals with gluten-related disorders.

From an agronomic perspective, buckwheat exhibits remarkable adaptability to diverse agroecological conditions, including marginal soils and variable climates, while requiring minimal agricultural inputs. This characteristic enhances its relevance within the context of sustainable agriculture and climate-resilient cropping systems. Furthermore, emerging evidence supports its use not only in food systems but also in the bio-based materials sector—particularly in the formulation of biodegradable packaging films using buckwheat-derived starch.

Despite its promising functional properties, the industrial-scale utilization of buckwheat starch in biodegradable food packaging remains underdeveloped. Although preliminary research has established the potential of buckwheat starch to form films with desirable barrier, antimicrobial, and mechanical characteristics, these outcomes are largely achieved through various modification strategies, including chemical crosslinking with organic acids (e.g., citric or tartaric acid), incorporation of essential oils, ultrasonic treatment, and thermal processing. However, significant technological challenges continue to hinder its widespread application. These include intrinsic limitations such as high hydrophilicity leading to poor water resistance, insufficient tensile strength, low elongation capacity, and inadequate structural flexibility that complicate reproducibility and scalability in industrial contexts. Future research should prioritize the standardization of buckwheat starch extraction and modification protocols, aiming to advance the development of high-performance, scalable, and environmentally sustainable buckwheat starch-based food packaging materials.

## Figures and Tables

**Table 1:** Proximate composition of common buckwheat and Tartary buckwheat, adapted and modified from [25,31,50,52,53,54,55].

Parameters	Tartary Buckwheat	Common Buckwheat
Ash (%)	1.8–2.70	1.53–2.50
Proteins (%)	5.7–16.4	8.51–18.57
Lipids (%)	3–4.7	1.5–4
Total dietary fibers (%)	6.1–8.4	8.4–10.0
Starch (%)	60.0–70.2	59–69
Carbohydrates (%)	55.4–57.40	54.50–73.6

## Data Availability

Not applicable.
